# ASSOCIATION OF PERSISTENT DEPRESSIVE SYMPTOMS WITH PHYSICAL ACTIVITY IN KNEE OSTEOARTHRITIS

**DOI:** 10.1093/geroni/igac059.2597

**Published:** 2022-12-20

**Authors:** Rhea Mehta, Michelle Shardell, Alice Ryan, Yu Dong, Brock Beamer, Marc Hochberg, Alan Rathbun

**Affiliations:** University of Maryland, Baltimore, Baltimore, Maryland, United States; University of Maryland School of Medicine, Baltimore, Maryland, United States; University of Maryland Baltimore, Baltimore, Maryland, United States; University of Maryland, Baltimore, Baltimore, Maryland, United States; University of Maryland Baltimore, Baltimore, Maryland, United States; University of Maryland, Baltimore, Baltimore, Maryland, United States; University of Maryland, Baltimore, Baltimore, Maryland, United States

## Abstract

Comorbid depression in knee osteoarthritis (OA) is associated with declines in physical activity, but how persistent depressive symptoms impact physical activity over time remains unclear. We aimed to determine how the persistence of depressive symptoms affects physical activity in knee OA. Participants (n=2,222) from the Osteoarthritis Initiative had radiographic disease (Kellgren-Lawrence grade ≥ 2) in at least one knee. The Center for Epidemiologic Studies Depression Scale (CES-D; range = 0-60) assessed depressive symptoms from baseline through the first three annual follow-up visits, and persistence was operationalized using the cumulative average severity of symptoms over time. Self-reported physical activity was measured from the first to fourth annual follow-up visit using the Physical Activity Scale for the Elderly (PASE; range = 0-793). The primary method of analysis utilized marginal structural models and included exposure by time interactions in the structural outcome model. Baseline depressive symptoms negatively impacted physical activity at the first follow-up (β = -0.7279; 95% CI: -1.1645, -0.2912), but at later time points, effect estimates were closer to the null and not statistically significant. The association between time-averaged CES-D scores from baseline through the first follow-up and physical activity at year two was -0.1410 (95% CI: -0.7105, 0.4286); and 0.2578 (95% CI: -0.3261, 0.8415) for average CES-D scores through follow-up visit two and physical activity at year three. Thus, the negative influence of persistent depressive symptoms on physical activity decreased over time. Physical activity may not consistently decline with persistent depressive symptoms in adults with knee OA.

